# Prevention of Lysosomal Storage Diseases and Derivation of Mutant Stem Cell Lines by Preimplantation Genetic Diagnosis

**DOI:** 10.1155/2012/797342

**Published:** 2012-12-26

**Authors:** Gheona Altarescu, Rachel Beeri, Rachel Eiges, Silvina Epsztejn-Litman, Talia Eldar-Geva, Deborah Elstein, Ari Zimran, Ehud J. Margalioth, Ephrat Levy-Lahad, Paul Renbaum

**Affiliations:** ^1^“ZOHAR” PGD Laboratory, Medical Genetics Institute, Shaare Zedek Medical Center, P.O. Box 3235, Jerusalem 91031, Israel; ^2^School of Medicine, The Hebrew University, Jerusalem, Israel; ^3^Stem Cell Laboratory, Medical Genetics Institute, Shaare Zedek Medical Center, P.O. Box 3235, Jerusalem 91031, Israel; ^4^IVF Unit, Shaare Zedek Medical Center, P.O. Box 3235, Jerusalem 91031, Israel; ^5^Gaucher Clinic, Shaare Zedek Medical Center, P.O. Box 3235, Jerusalem 91031, Israel

## Abstract

Preimplantation genetic diagnosis (PGD) allows birth of unaffected children for couples at risk for a genetic disorder. We present the strategy and outcome of PGD for four lysosomal storage disorders (LSD): Tay-Sachs disease (TSD), Gaucher disease (GD), Fabry disease (FD), and Hunter syndrome (HS), and subsequent development of stem cell lines. For each disease, we developed a family-specific fluorescent multiplex single-cell PCR protocol that included the familial mutation and informative markers surrounding the mutation. Embryo biopsy and PGD analysis were performed on either oocytes (polar bodies one and two) or on single blastomeres from a six-cell embryo. We treated twenty families carrying mutations in these lysosomal storage disorders, including 3 couples requiring simultaneous analysis for two disorders (TSD/GD, TSD/balanced Robertsonian translocation 45XYder(21;14), and HS/oculocutaneus albinism). These analyses led to an overall pregnancy rate/embryo transfer of 38% and the birth of 20 unaffected children from 17 families. We have found that PGD for lysosomal disorders is a safe and effective method to prevent birth of affected children. In addition, by using mutant embryos for the derivation of stem cell lines, we have successfully established GD and HS hESC lines for use as valuable models in LSD research.

## 1. Introduction

Preimplantation genetic diagnosis (PGD) allows genetic diagnosis of embryos in very early stages, with the purpose of avoiding the transmission of genetic diseases to offspring. PGD represents an alternative to prenatal diagnosis and termination of pregnancy, in couples at risk of transmitting these disorders. Since the first PGD application [1], it has been made available for a large number of rare genetic disorders and the number of cycles increase yearly [2]. PGD for molecular diseases is challenging for two reasons: the first is the necessity to analyze the disorder at the single cell level, and the second is due the need to build unique multiplex polymerase chain reaction (PCR) protocols for each specific mutation of interest and each family. The PGD protocol must allow reliable results in a short period of time (between 24 and 72 hours) so that embryos can be transferred within the window of successful implantation. It is also possible to combine multiple PCR-based assays on single cells, which permits the analysis of two or more indications at once, for example, testing two single gene disorders in the same couple [3, 4].

Although, in theory, PGD could be accomplished using mutation analysis alone, this would be accompanied by a high error rate due to allele drop out (ADO) [5] which can reach 20% [6]. ADO results from preferential allele-specific amplification and can lead to misinterpretation of the genetic status of the oocyte/embryo [7]. Therefore, PGD protocols need to include several linked informative polymorphic markers flanking the disease gene in order to minimize misdiagnosis due to ADO, allowing accuracy rates approaching 100% [8]. To accomplish this, prior to the PGD cycle, a family haplotype is built based on genomic DNA from immediate family members and relatives to establish linkage between the mutation and informative microsatellite markers [9].

Most protocols use blastomere biopsy (single cells from a 6–8 cell, day 3 embryo) for embryo diagnosis. PGD using polar bodies (extruded by the oocytes) analysis has been shown to be an effective method for maternal autosomal dominant, X-linked, and, in some cases, recessive disorders [10].

Basic research for human disease is commonly studied in animal and cellular model systems. For many genetic disorders these models are not available. In these cases, diseased embryos obtained through PGD can provide a novel resource for the derivation of mutant human embryonic stem cell (HESC) lines. Such cell lines may be induced to differentiate different tissues and can be exceptionally useful for basic research studies as well as for drug screening and development.

Many clinicians are not aware of the new genetic methods for diagnosis and prevention of inherited lysosomal disorders. We present the development of PGD protocols for four lysosomal storage disorders: Tay Sachs, Gaucher type I, Fabry, and Hunter syndrome; two of them, Tay Sachs and Gaucher, have a very high carrier rate in the Ashkenazi Jewish population [11].

Tay Sachs (TSD, GM2 gangliosidosis type B, OMIM #272800) is a recessive neurodegenerative lysosomal storage disease caused by Hexosaminidase A isoenzyme deficiency. The most severe form of the disease is characterized by rapid progressive neurodegeneration, developmental regression, and death during age of 3-4 years [12]. Tay Sachs is found with a 10-fold higher frequency in the Ashkenazi Jewish population (1 : 3600 versus 1 : 360000 in general non-Jewish population) [11, 13, 14]. Identification of the deficient enzyme opened the doors to screening programs in the Ashkenazi population allowing the detection of carriers which are at high risk for an affected child. Three common mutations were identified in the Hexosaminidase A gene, *HEXA*, among Ashkenazi Jews: a 4 bp (basepair) insertion in exon 11 (accounting for 70% of the mutations), a splice mutation in intron 12 IVS12G>C, and and a G>A nucleotide substitution in exon 7 causing p.Gly269Ser [13].

Gaucher disease (GD, OMIM #230800), the most common lysosomal storage disorder (Goldblatt [15]), is caused by Glucocerebrosidase deficiency. Mutations in the *GBA* gene are also highly prevalent in Ashkenazi Jewish population (type I GD) with an affected allele frequency of 0.035 [14]. Among the three types of GD, type I nonneuronopathic is the most prevalent and the GBA c.1226A>G mutation is the most frequent mutation accounting for 75% of all diseases causing alleles [16]. Most patients who are homozygous for the c.1226A>G mutation are asymptomatic but compound heterozygotes between c.1226A>G and other mutations can cause characteristic symptoms of Gaucher disease (organomegaly, anemia, bone involvement) and require life enzymatic replacement treatment [11]. 

Mucopolysaccharidosis type II (MPS II) or Hunter syndrome is a lysosomal storage disease caused by a deficiency of the enzyme iduronate-2-sulfatase (*IDS*) [17]. It is an X-linked disorder manifesting in males and rarely in females [18]. There are two different types of Hunter/MPS II, classified by their age of onset and the severity of the symptoms: the early onset type is characterized by more severe symptoms and the late-onset form is characterized by milder symptoms. Hunter/MPS II is estimated to affect one from 100,000 to 150,000 births worldwide [19]. Symptoms of both forms of this disorder include coarse facial features, a large head, stiff joints, hearing loss, increased hair production, enlarged liver and spleen, and carpel tunnel syndrome [17] (Wraith). The early onset form of Hunter/MPS II manifests shortly after two years of age, and additional symptoms include mental deterioration, mental retardation, aggression, and hyperactivity sometimes leading to death [20].

Fabry disease is caused by an X-linked (dominant) error of metabolism wherein deficiency of the lysosomal enzyme *α*-galactosidase A results in progressive deposition of glycosphingolipids, predominantly globotriaosylceramide [21], in renal glomerular and tubular epithelial cells, myocardial cells, heart valve fibrocytes, neurons of dorsal root ganglia, and in endothelial smooth cells of blood vessels [22]. Hence, Fabry disease is marked by multisystemic disease but considerable phenotypic variability exists possibly because of molecular heterogeneity [23]; most families carry a private mutation [24].

We present 20 families who underwent preimplantation diagnosis for prevention of birth of children with inherited lysosomal disorders, without the need for invasive prenatal diagnosis procedure and termination of pregnancy. These methods are generally applicable for any disorder in which the genetic basis of the disease is known.

## 2. Materials and Methods

### 2.1. Patients

#### 2.1.1. Tay Sachs

Of the eleven TS families that presented to our clinic, one of them was a double-carrier for Tay Sachs and Gaucher type 1, and in another, the male in addition to being a carrier of Tay Sachs was also a carrier of a balanced robertsonian translocation 45XYder(21;14). Details of the patients are presented in Table 1(a). Nine out of eleven families knew about their carriership status from screening tests performed prior to the first pregnancy. Four childless families decided to perform PGD rather than prenatal diagnosis and five families had previously performed prenatal diagnosis resulting in one termination of pregnancy due to an affected embryo.

#### 2.1.2. Gaucher Disease Type 1

Four GD families (including a couple also carrying TS mutations; Family number 1 (Tables 1(a) and 1(b)) presented to our PGD unit. Couple number 2 lost their only daughter affected with GD at the age of five. The daughter was a compound heterozygote for the common N370S and a private paternal inherited R359Q mutation. She presented in the first year of life with hepatosplenomegaly and developed severe pulmonary involvement at age of 3 years. Despite high-dose enzyme replacement therapy she required pulmonary transplant at the age of 4 and died at the age of 5 of lung related complications. In couple number 3 (Table 1(b)) the female was affected with Gaucher disease (N370S/N370S) and the husband was a carrier of the 84GG mutation, resulting in a 50% chance of an affected embryo predicted to have a severe phenotype.

#### 2.1.3. Fabry Disease

Two couples in which males were affected with Fabry disease presented to our clinic. In one of the couples, he also was affected with nonobstructive azoospermia (NOA) requiring micro-TESE for in vitro fertilization. Since in both couples the males were affected and since Fabry is an X-linked disorder, all the male embryos will be healthy while all the female embryos will be carriers. Since seventy percent of female carriers of Fabry disease manifest clinical symptoms during life (ref), both our couples chose not to transfer carriers.

#### 2.1.4. Mucopolysaccharidosis Type II (Hunter Syndrome)

Three HS couples presented to our clinic (Table 1(c)). In 2 couples, the females were sisters aged 34 and 25 years and were carriers of a missense p.L410P mutation in the iduronate 2-sulfatase gene (IDS). They had a brother who had died in childhood of Hunter syndrome. Both sisters had previously delivered healthy children after chorionic villous sampling (CVS) testing to rule out an affected fetus. In the third couple, the female was a carrier of a deletion of exons 4–7; she had two brothers who had died of Hunter syndrome. This couple had previously performed prenatal analysis and pregnancy termination due to the identification of an affected male fetus. This couple was also found to be carrying p.G47D and p.R402Q mutations in the *TYR* gene (based on a relative of the proband who had suffered from oculocutaneus albinism and subsequent genetic testing of both members of the couple). For this couple, a double PGD for Hunter syndrome and oculocutaneus albinism was performed.

### 2.2. IVF-Ovarian Stimulation, Oocyte Retrieval, Fertilization, and Biopsy

Controlled ovarian stimulation and IVF treatment were performed using the standard long downregulation protocol. For frozen-thawed embryo cycles, endometrium preparation was performed using oral estradiol valerate (Estrofem 4–8 mg daily (Novo Nordisk, Denmark)) and vaginal Utrogestan (micronized progesterone 900 mg/daily (CTS, France)). Polar body and blastomere (one cell of a six—eight cell embryo) biopsy, ICSI, and embryo cultures were performed as previously described [9, 25]. 

### 2.3. Molecular Analysis

DNA was extracted from peripheral blood cells from couples, affected children, and first-degree family members. For each disease, polymorphic microsatellite markers surrounding the diseased gene were identified and informative markers were used construct haplotypes in each family. These markers and the family mutations were then used for the development of single-cell multiplex assays. The panels of markers used for PGD analysis of each disease are presented in Table 2. A multiplex-nested PCR reaction was used as previously described [8]. Only samples that were informative for a minimum of three polymorphic markers flanking the mutation were considered for diagnosis. Stringent precautions to avoid any source of contamination, as recommended by the European Society for Human Reproduction and Embryology (ESHRE) PGD consortium, were used during all steps [26].

### 2.4. HESC Derivation and Maintenance

Derivation and maintenance of undifferentiated Shaare Zedek (SZ) Hunter and Gaucher cells were carried out according to routinely applied protocols on blastocysts diagnosed as mutant [27]. Spontaneous differentiation in vitro, DNA extraction and genotyping, RNA extractions and marker expression, chromosome analysis, and biochemical analysis were performed as previously described [27].

## 3. Results

A total of 20 families carrying mutations in four genes causing lysosomal diseases presented to our PGD unit. We performed 56 PGD cycles and analyzed 329 oocytes/embryos with an overall pregnancy rate of 38%. All but three couples have so far delivered healthy children. The results and intricacies of these analyses are described below, a summary of the PGD cycles is presented in Table 3.

### 3.1. PGD for Tay Sachs

All eleven families were of Ashkenazi Jewish origin and carried one of the three common mutations in the HEXA gene c.1278insTATC, IVS12G>C, or p.Gly269Ser. Nine families achieved pregnancies and delivered between one and two (twin) healthy babies per cycle. Couple number 1, carried both TS and GD mutations necessitating analysis of both loci, they had three children from three separate PGD cycles. In couple number 3, of 10 screened microsatellite markers upstream from the HEXA gene, none were informative for the maternal wild-type alleles, and therefore only the wild-type paternal allele could be used to identify unaffected embryos. Only one embryo was transferred in one PGD cycle although no pregnancy was achieved. In couple number 7, of 16 microsatellite markers screened, only two were found to be fully informative for blastomere analysis, and since the partners presented different mutations, the mutations could not be used in blastomere analysis because of possible misdiagnosis due to ADO. However, by using polar body analysis (only maternal alleles are analyzed), six informative maternal markers were identified and used in one PGD cycle that did not yet result in pregnancy. Couple number 11, in which the male was also carrier of a balanced translocation, underwent three PGD cycles. In a total of 24 embryos analyzed, only three were found to be balanced/normal for the paternal translocation and nonaffected with TS, no pregnancy was achieved in two separate transfers. None of the couples agreed to perform prenatal confirmation by CVS or amniocentesis but all babies were tested for TS familial mutations after birth and were found to be unaffected and in accordance with the embryos transferred (wild type or carriers). A total number of 27 PGD cycles were performed with a pregnancy rate (per embryo transfer) of 40% and 11 healthy babies were born.

### 3.2. PGD for Gaucher Disease Type 1

Four Couples who were carriers of GS mutations underwent PGD and all families achieved healthy children. Family number 1, carriers of both TS and GD IVS2+1G>A/N370S mutations, was described above and had three healthy children through PGD. Couple number 2 was heterozygotes for two different mutations (p.N370S and p.R359Q) and underwent 11 PGD cycles. One cycle resulted in a spontaneous abortion in week 10, another in the birth of a healthy boy, and the most recent cycle resulted in an ongoing twin pregnancy in week 25. In Couple number 3, the female was homozygous for Gaucher disease type 1 (N370S/N370S) and the husband was a carrier of the 84GG mutation, therefore all embryos will at least be obligatory carriers of the maternal mutation. In one cycle of 7 embryos, four were found to be carriers and two of these were transferred resulting in birth of a healthy child. The wild-type paternal allele was confirmed on genomic DNA of the baby after birth. In family number 4, of six embryos two were found to be wild type and the remainder were mutant. One healthy child was born from this PGD cycle and the wild-type status was confirmed after birth. 

### 3.3. PGD for Hunter Syndrome

Three couples presented to our center with HS and all had healthy babies. Couples number 1 and number 2 (sisters carrying the L410P mutation in the IDS gene) underwent four and one (resp.) PGD cycles. Since Hunter syndrome is an X-linked disorder and the females in both couples were carriers, indirect oocyte analysis by polar bodies 1 and 2 is sufficient for accurate diagnosis and was performed in these cases. Couple number 1 delivered a singleton and couple number 2 had twins. In couple number 3, the female was a carrier of a deletion of exons 4–7 in the IDS gene, and both members of the couple were also carriers of different mutations in the TYR gene, requiring simultaneous PGD for both Hunter syndrome and oculocutaneus albinism (a recessive disorder). Since in cases of autosomal recessive disorders, the information of both maternal and paternal contribution is required for complete diagnosis, a single blastomere was biopsied and used for PGD analysis for both diseases. This couple underwent seven PGD cycles, resulting in birth of healthy twins from a combined fresh and frozen blastomere analysis. 

### 3.4. PGD for Fabry Disease

In both couples analyzed for FD, the males were affected, and since Fabry is an X-linked disorder, all male embryos are expected to be healthy; furthermore, 70% female carriers show clinical manifestations, we therefore used sex selection as a means to select for disease free embryos. Couple #1 underwent three PGD cycles. The male in this couple had nonobstructive azoospermia (NOA) requiring micro-testicular sperm extraction (TESE) for in vitro fertilization (IVF). Three cycles lead to a singleton pregnancy and subsequent birth of a healthy boy. Couple number 2 underwent one PGD cycle; analysis of two embryos led to the diagnosis of one male. Transfer of this embryo resulted in the birth of a healthy boy.

### 3.5. Derivation of Stem Cell Lines

**Figure 1:**
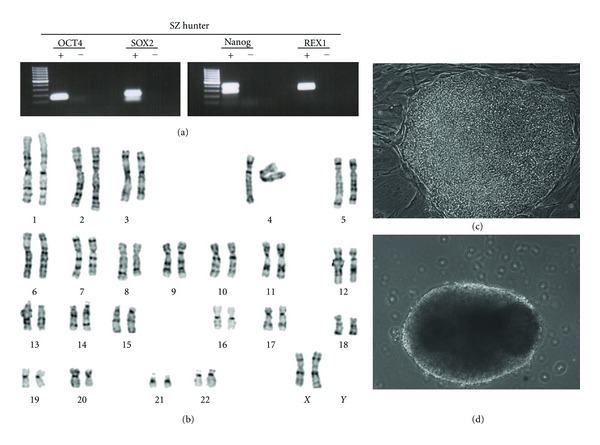
Characterization of SZ-Hunter HESCs for the expression of undifferentiated cell-specific markers, karyotype, and pluripotent potential. (a) RT-PCR products for the undifferentiated gene-specific markers OCT4, SOX2, NANOG, and REX-1, using cDNA-specific primers, in undifferentiated SZ-Hunter HESCs at passage P17. (b) Karyotype analysis for SZ-Hunter cells by Giemsa staining. (c) A typical morphology of an undifferentiated SZ-Hunter HESC colony. (d) A cystic embryoid body (EB) established from SZ-Hunter HESCs, grown for 20 days in culture of suspension.

Mutant embryos from these 4 lysosomal diseases (after IRB approval and family consent) were donated for stem cell line derivation. Out of 28 embryos, two HESC lines were obtained (Table 4), one from a female mutant Hunter embryo and one from a compound heterozygote 84GG/N370S for Gaucher disease. These new lines display characteristics typical of HESCs, expressing a panel of undifferentiated markers, including NANOG, OCT4, SOX2, and REX (Figure 1(a)). Chromosomal analysis by Giemsa staining, carried out on metaphase spreads at passage 8, showed a normal 46XX human karyotype for Hunter cell line (Figure 1(b)) and 46XY for the Gaucher line. HESC colonies and embryoid bodies were observed as well (Figures 1(c) and 1(d)). 

## 4. Discussion

We present a reliable method for preventing birth of affected children for parents carrying mutations in genes causing lysosomal disorders and subsequent development of stem cell lines for research in the field of lysosomal diseases. When couples who are carriers of a genetic disorder wish to conceive they have the option to either not perform prenatal genetic testing and to accept the possibility of an affected child or to perform an invasive test (chorionic villus sampling or amniocentesis) in order to determine the genetic status of the embryo. Both of these invasive methods are accompanied by a small risk of abortion due to the procedure [28]. Furthermore, at present, these tests cannot be performed earlier than 10-11 weeks of gestation, therefore a significant period of time remains between the date the pregnancy is detected until the results of genetic testing are received causing increased anxiety and uncertainty in respect to the future of the pregnancy. Out of more than 50 lysosomal diseases known, most lysosomal storage disorders cause a severe phenotype and have a high recurrence risk of 25%. Although some treatment is available today for a few of these diseases (Gaucher disease, Fabry disease, Hunter syndrome, Pompe syndrome, etc.), the efficacy varies for different disorders, the drugs (enzyme replacement therapies) are given intravenously and do not pass blood brain barrier, therefore they are less effective for the diseases with neurological involvement. Still there is a large number of lysosomal disorders that have no available treatment today and cause death at an early age (Tay Sachs, Krabbe disease, etc.).

PGD is a technique that offers an alternative to pregnancy termination of an affected embryo by analyzing single blastomeres of biopsied embryos or polar bodies 1 and 2 from oocytes, and returning to the uterus only unaffected embryos. Since PGD was first performed in 1991 [1], by using different strategies, the technique has become very accurate with a misdiagnosis rate of less than 1% [26].

Screening tests for Tay Sachs disease prior to, or at the beginning of, pregnancy is performed free of charge in Israel for all Ashkenazi Jewish couples. All our couples found that both partners were carriers during the screening tests and while some approached our unit immediately for PGD, others performed CVS. One couple performed a pregnancy termination of an affected embryo prior to their arrival at our PGD clinic.

Since Gaucher disease can be asymptomatic in Ashkenazi Jews (who most commonly carry the N370S mutation), we most commonly perform PGD for this lysosomal disorder only in cases in which the phenotype is predicted to be severe based on the mutations carried by the couple. In the first family the couple were carriers of both Tay Sachs and Gaucher disease mutations and the main reason for PGD was Tay Sachs, a lethal disease, even though the Gaucher mutation combination of IVS2+1G>A/N370S predicted a possible more severe phenotype. In both couples number 2 and number 4 one of the partners was a carrier for the 84GG mutation predicting a possible severe phenotype. In couple number 3 the male was carrier of a private mutation R359Q that in combination with the N370S caused a severe pulmonary disease in their daughter leading to death at the age of 5 years despite high-dose enzyme replacement therapy. In the case of Hunter syndrome, the two sisters carrying mutations in the IDS gene had a brother who died of the disease. Both women had prior pregnancies and performed CVS testing prior to the PGD cycles. The third couple came to our PGD clinic after performing a termination of pregnancy due to an affected male embryo detected in prenatal testing.

In both couples with Fabry disease the male partners were affected, and since inheritance of this disease is X-linked, it is predicted that all their female offspring will be carriers and all males unaffected. One of these males also had severe oligospermia requiring IVF irrespective of PGD. In the second couple there was no known infertility but since more than half of female carriers of Fabry disease develop symptoms during life [22], they preferred medical sex selection for males.

The mutant embryos derived from PGD are a valuable resource for disease research due to the possibility of developing mutant stem cell lines. In our unit, mutant embryos that are donated by the family for stem cell research undergo culturing for stem cell line development. Out of these four lysosomal disorders we have successfully established 2 lines. One Gaucher stem cell line was derived from mutant embryos caring 84GG and N370S mutations is of particular of interest due to recent evidence of correlations between Parkinson disease and Gaucher [29]. In addition, a mutation carrying Hunter line was derived from a female embryo, but again, since Hunter disease is an X-linked disorder, mutant males present a phenotype while most females are asymptomatic carriers. Although our line presented all the characteristic markers of a pluripotent stem cell line it may be less appropriate for research since it is derived from a female embryo. 

In summary, we have presented our PGD experience for 20 families with Tay Sachs, Gaucher, Fabry diseases, and Hunter syndrome. In 56 PGD cycles, 329 oocytes/embryos were analyzed, which led to the birth of 20 unaffected children, with an overall pregnancy rate of 38% per embryo transfer (the overall pregnancy rate reported in the ESHRE XI data collection was 27%). Surprisingly, we observed a significantly higher mutant embryo rate than we expected. In autosomal recessive disorders only 25% of embryos are expected to be mutant, however, of the 166 embryos we analyzed for Tay Sachs, 65 (39%) were mutant and not transferable, and analysis of embryos from Gaucher carrier couples yielded 33/66 (50%) mutant embryos. Inheritance of X-linked disorders expected to yield 50% wild-type offsprings, 25% carriers (females), and 25% affected males. In the case of Hunter and Fabry diseases, since carrier females are known to show some disease manifestation, these embryos are not transferred either. Embryo analysis of the X-linked disorders in our study yielded 53/72 (74%) mutant or carrier embryos in Hunter disease, and 18/25 (72%) nontransferrable embryos in Fabry couples. While we are not able to offer an explanation for the skewing towards mutant embryos observed here, these statistics confirm the importance of the role PGD has played in family planning for these couples.

Of the 20 unaffected children born, fourteen births were singletons and three were twins, the wild type or carrier disease status was confirmed in all cases after birth. Four unaffected children were born in families where PGD needed to be performed for 2 different disorders simultaneously, a circumstance which significantly lowers the number of unaffected embryos available for transfer. At present, of these 20 families, 17 have given birth to unaffected children (2 of the remaining 3 families have only undergone 1 PGD cycle), thus far the take-home baby rate per family in this study was 85%.

## Figures and Tables

**Table:** (a)

Family number	Mutation female/male	Healthy/affected children prior to PGD	Female age (years)
1	IVS12G>C/Gly269Ser	0	25
2	1278insTATC/1278insTATC	0	32
3	1278insTATC/1278insTATC	1/0	29
4	1278insTATC/Gly269Ser	1/0	31
5	1278insTATC/1278insTATC	3/0	33
6	1278insTATC/Gly269Ser	1/0	30
7	1278insTATC/Gly269Ser	0	31
8	1278insTATC/1278insTATC	3/0	34
9	IVS12G>C/Gly269Ser	0	28
10	1278insTATC/1278insTATC	2/TOP*	33
11	1278insTATC/1278insTATC	0/1**	29

TOP*: termination of pregnancy due to affected embryo.

**The male was also a carrier of a balanced Robertsonian translocation 45XYder(21;14).

**Table:** (b)

Family number	Mutation female/male	Healthy/affected children prior to PGD	Female age (years)
1	IVS2+1G>A/N370S	0	25
2	N370S/R359Q	0/1*	34
3	N370 homozygous/84GG	1/0	29
4	Arg496His/84GG	0	30

*The daughter died at the age of five due to severe pulmonary involvement.

**Table:** (c)

Family number	Mutation female	Healthy/affected children prior to PGD	Female age (years)
1	L410P*	2/0	34
2	L410P*	1/0	25
3	Del exons 4–7	0/TOP*	24

TOP*: termination of pregnancy due to affected embryo.

**Table 2:** Polymorphic microsatellite markers used in PGD/LSD analyses.

Disease	Markers
Tay Sachs	D15S204, D15S110, D15S197, TS-AT3 (chr15: 70157048-70157297), TS-TATT (chr15: 70231314-70231376), TS-TTTC (chr15: 70404391-70404690), TS-TC (chr15: 70408504-70408653), TS-ATCT (chr15: 70417649-70417798), TS-CA (chr15: 70420187-70420336), TS-TA (chr15: 70465534-70465733), TS-TG (chr15: 70172306-70172338), D15S215, D15S188, D15S169, D15S818

Gaucher disease type 1	D1S2715, D1S2858, D1S305, Gau-GT2 (chr1: 153399641-153399790), Gau-AC (chr1: 153726984-153727183), Gau-GT (chr1: 153425519-153425668), Gau-TTAT (chr1: 153461596-153461795), Gau-AAAG (chr1: 153462605-153462854), Gau-AAT (chr1: 153480717-153480916), Gau-TAT (chr1: 153519755-153519904), GAu-AAT2, (chr1: 153527305-153527554), D1S1153, Gau-AC3 (chr1: 153593444-153593643), D1S27777, Gau-AC4 (chr1: 153897882-153898031), D1S303, D1S2140, D1S2721, D1S2624

Mucopolysaccharidosis II	DXS731, DXS1215, DXS1691, DXS8091, DXS6687, DXS2496, DXS1185, DXS1193, DXS457, DXS1123, Hunter-GAGG (chrX: 148394342-148394541), Hunter-AC (chrX: 148405052-148405251), DXS8086, DXS8377, DXS8069, DXS7423, DXS8011

Fabry disease (sex selection markers)	AmelogB96, SRY, DXS1254, DXS998, DXS1215, DXYS154, DXS566, DXS8377

Polymorphic microsatellite markers used in PGD/LSD analyses: location of markers is based on human USCS genome browser assembly March 2006, NCBI36/hg18 presented in the order of location on the chromosome (http://genome.ucsc.edu/cgi-bin/hgGateway?hgsid=319485427&clade=mammal&org=Human&db=hg18).

The markers that do not have standard DS nomenclature were named arbitrarily and their physical chromosome and basepair location are indicated.

**Table 3:** IVF-PGD treatment outcome.

Disease	Family number	Total cycles	Number of oocytes retrieved	Number of oocytes/embryos biopsied	Wild type/ carrier/mutant^#^	Number of transferable embryos	Number of embryos transferred/cycle	Treatment outcome
	1*	3	32	26	3/4/17	7	2	3 children from 3 cycles
	2	3	16	16	8/4/4	12	2	Twins
	3	1	5	4	0/1/3	1	1	No pregnancy
	4	2	15	13	4/5/4	7	2	Twins
	5	1	12	10	3/7/0	10	2	One child
Tay Sachs	6	7	44	38	10/20/8	30	1-2	One child
	7	1	5	5	2/0/3	2	2	No pregnancy
	8	4	26	18	4/7/3	11	1-2	One child
	9	1	11	10	2/5/3	7	1	One child
	10	1	8	6	0/4/2	4	1	One child
	11**	3	30	24	3/8/13	3	3	No pregnancy

Gaucher	2	11	28	23	6/10/7	16	2-3	One spontaneous abortion week 10, one child, and one ongoing twin pregnancy week 22
	3	2	15	13	0/6/7	6	1-2	One child
	4	1	6	6	2/2/2	4	1	One child

	1	4	45	35	6/10/19	6	1-2	One child
Hunter	2	1	16	11	4/0/5	4	2	Twins
	3***	7	35	33	9/0/19	9	1-2	Twins

Fabry	1	3	38	23	6/0/17	7	1-2	One child
	2	1	4	2	1/0/1	1	1	One child

^#^Only embryos/oocytes which were fertilized and definitively diagnosed are included.

*Double carriers for Tay Sachs and Gaucher disease.

**The male was also a carrier of a balanced robertsonian translocation 45XYder(21;14), therefore some embryos could not be transferred due to unbalanced karyotype.

***Female carrier of mutation in IDS gene; both partners are carriers of mutations in the Tyrosinase (*TYR*) gene.

**Table 4:** Derivation of stem cell lines.

Disease	Number of mutant embryos received	Embryos plated	Cell line
Gaucher	2	No	No
Gaucher	2	No	No
Gaucher	4	4	1
Hunter	15	8	1
Tay Sachs	5	3	No

Total	28	15 (53.6%)	2 (13.4%)*

*Per embryos plated.
